# Even harmonic generation in isotropic media of dissociating homonuclear molecules

**DOI:** 10.1038/srep32653

**Published:** 2016-09-06

**Authors:** R. E. F. Silva, P. Rivière, F. Morales, O. Smirnova, M. Ivanov, F. Martín

**Affiliations:** 1 Departamento de Química, Universidad Autónoma de Madrid, 28049 Madrid, Spain; 2 Max-Born-Institut, Max Born Strasse 2A, D-12489 Berlin, Germany; 3 Department of Physics, Voronezh State University, Universitetskaya pl., 1, Voronezh, Russia, 394036.; 4 Instituto Madrileño de Estudios Avanzados en Nanociencia, 28049 Madrid, Spain; 5 Condensed Matter Physics Center (IFIMAC), Universidad Autónoma de Madrid, 28049 Madrid, Spain

## Abstract

Isotropic gases irradiated by long pulses of intense IR light can generate very high harmonics of the incident field. It is generally accepted that, due to the symmetry of the generating medium, be it an atomic or an isotropic molecular gas, only odd harmonics of the driving field can be produced. Here we show how the interplay of electronic and nuclear dynamics can lead to a marked breakdown of this standard picture: a substantial part of the harmonic spectrum can consist of *even* rather than odd harmonics. We demonstrate the effect using ab-initio solutions of the time-dependent Schrödinger equation for 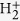 and its isotopes in full dimensionality. By means of a simple analytical model, we identify its physical origin, which is the appearance of a permanent dipole moment in dissociating homonuclear molecules, caused by light-induced localization of the electric charge during dissociation. The effect arises for sufficiently long laser pulses and the region of the spectrum where even harmonics are produced is controlled by pulse duration. Our results (i) show how the interplay of femtosecond nuclear and attosecond electronic dynamics, which affects the charge flow inside the dissociating molecule, is reflected in the nonlinear response, and (ii) force one to augment standard selection rules found in nonlinear optics textbooks by considering light-induced modifications of the medium during the generation process.

Attosecond technology originated in nonlinear optics, with high harmonic generation (HHG) being the fundamental physical process underlying the generation of attosecond pulses^1^,^2^. In the two decades since its inception, attosecond science has grown rapidly^3^,^4^,^5^ with applications in physics, chemistry^6^,^7^,^8^, materials science^9^,^10^,^11^ and even biology^12^,^13^. High harmonic emission results from nonlinear response of a medium to an intense laser field. Its basic mechanism was first described in refs 14, 15, 16 (see also ref. 17). After the intense laser field frees an electron from the ionic core, the electron gains energy from the field and revisits the parent ion. Radiative recombination converts the gained energy into high-frequency radiation.

In isotropic atomic gases irradiated by long IR pulses, the electron round-trips between ionization and recombination are launched during successive laser half-cycles. Mirror symmetry of the driving electric field implies that these round trips are mirror images of each other. Electrons revisiting the parent ion from opposite directions yield emission bursts with the same amplitude but opposite signs. As a consequence, even-order harmonics interfere destructively and vanish, while the odd-order harmonics interfere constructively^18^, leading to the spectral peaks at odd multiples of the fundamental frequency, Ω_*n*_ = (2*n* + 1)*ħω*_IR_.

A similar behavior is commonly expected for any isotropic medium, such as an isotropic distribution of homonuclear diatomic molecules. However, the physical picture underlying high harmonic generation suggests that the expectation of odd-only harmonics requires that the inversion symmetry holds during the whole interaction. This is indeed the case if the molecular nuclei do not, or barely, move. However, as predicted in recent theoretical work on 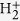
19,20,21,22, mostly using low dimensionality models^19^,^20^,^21^, new features may arise in the high harmonic spectrum if the nuclei move significantly during one or several laser half cycles. These include the reduction of the maximum (cutoff) energy in the harmonic spectrum^19^, a modest red shift of the harmonic peak positions^22^, suppression of specific odd harmonics^21^ and, as found more recently, the appearance of weak even harmonics between the strong odd harmonic peaks^20^.

Common wisdom suggests that even harmonics should disappear for long driving laser pulses, restoring the expected symmetry of the overall process and balancing contributions from the adjacent laser half-cycles. However, results of our study of high harmonic generation from 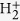, 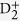 and 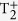, using a full dimensionality model for the electronic and vibrational degrees of freedom, contrast with this expectation even for rather long laser pulses. We find that the nuclear motion has an even more dramatic effect than anticipated in the previous work. For sufficiently long pulses, the HHG spectrum of the lighter molecules can exclusively consist of *even* harmonics in the plateau region. Furthermore, by changing the pulse duration, one can control the region of the plateau where the even harmonics appear. We unambiguously link the appearance of even harmonics to electron localization on one side of the molecule. This induces a permanent dipole during dissociation, even for pulses containing tens of laser cycles. The linear Stark effect associated with the permanent dipole introduces a relative phase between the two consecutive electron round trips initiated in the adjacent laser half-cycles. As this phase approaches *π*, the odd harmonics are replaced by the even ones.

Several works on even harmonic generation in diatomic molecules have been presented before^23^,^24^,^25^,^26^. However, the origin of even harmonic generation reported there is either the nuclear mass asymmetry^25^,^26^ or contributions from the nuclear quadrupole moment^23^,^24^. In contrast, here we deal with a homonuclear molecule and a purely dipole effect. When electron localization is present, it gives the strongest contribution to the even harmonics signal, on par with the strength of the odd harmonics. It is also important to emphasize that, in the present work, electron localization leading to even harmonic emission is purely induced by the IR field, at variance with earlier work^27^, where it results from the coherent superposition of gerade and ungerade molecular states arising from the interaction with a UV pump pulse that precedes IR irradiation.

## HHG Spectra

**Figure 1:**
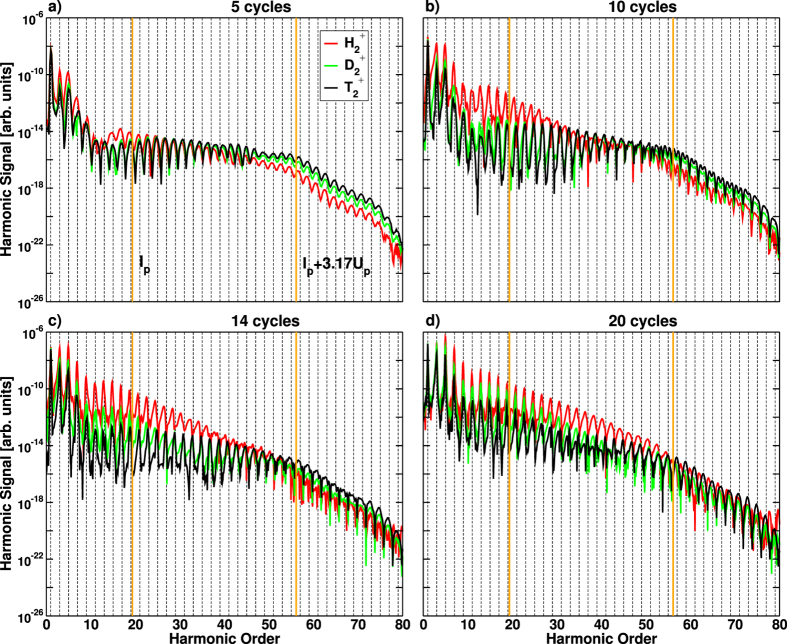
HHG spectrum for a pulse of 800 nm, *I* = 3 × 10^14^ W/cm^2^ and 5 (**a**), 10 (**b**), 14 (**c**) and 20 (**d**) optical cycles for 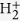, 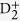 and 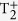. The dashed vertical lines indicates odd harmonics. The thick vertical lines indicate the ionization threshold (left line) and the cut-off energy obtained from *E*_cutoff_ = *I*_*p*_ + 3.17*U*_*p*_ (right line).

Figure 1 shows the calculated HHG spectra for the three different isotopes of the 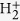 molecule and different pulse durations. For the shortest pulse, 5 optical cycles, our results are very close to our earlier results for low dimensionality models^20^,^21^. The spectra from different isotopes are generally very similar, with several broad even harmonic lines between orders 36–40. The effect is independent of the isotope and hence of the nuclear motion; it is associated with the frequency chirp induced by the changing laser intensity between successive half-cycles in an ultra-short pulse. As expected, the effect disappears for the longer, 10-cycle pulse, and for the heaviest isotope, where the nuclear motion is negligible. However, for the lightest isotope even harmonics remain very prominent for 10, 14, and 20-cycle pulses. As one moves to higher orders, the harmonic peaks in 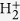 experience a red shift, up to a point that only even harmonics are observed for high enough orders. The spectral region where even harmonics dominate shifts to higher orders with increasing pulse duration. For the 20-cycle pulse even harmonics also appear for the 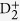 isotope. Additionally, for the longer pulses, the harmonics from 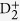 and 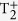 are strongly suppressed in the plateau region compared to 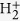, with rather dramatic modifications of the shape of the harmonic lines. What could be the origin of even harmonics in 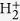, their shift with the pulse duration, and the emergence of two spectral regions where they are seen for 20-cycle pulse (the plateau and the cutoff)? What could be the origin of the combination of harmonic suppression and the dramatic harmonic line shape modifications for 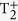
?

Two effects may be responsible. The first is electron localization, prominent in molecular dissociation^7^,^28^. Electron localization breaks the symmetry of the system and hence can lead to even harmonics^20^,^21^,^29^. The second is the asymmetry introduced by molecular dissociation between the raising and descending parts of the IR pulse^22^, in particular due to the shift in the characteristic ionization potential *I*_*p*_. As pointed out in ref. 22, this asymmetry leads to the red shift. The analysis of the harmonic spectra for different pulse durations and isotopes allows us to distinguish the contributions of these two effects. We argue that both are important but have quite different impact in multi-cycle pulses. While electron localization controls the interference of the emission bursts from successive laser half-cycles, the front-back asymmetry pertains to the longer time-scale and hence leads to finer-scale modifications in the harmonic spectrum, for long pulses.

We first analyze the impact of dissociation-induced asymmetry between the raising and the falling edges of the pulse. As discussed above, the harmonic spectrum is formed by the interference of emission bursts produced during successive half-cycles. If the system response during several successive half-cycles is nearly identical, odd harmonics will form during the raising part of the pulse. The same would apply at the falling edge of the pulse, even if the system has changed due to dissociation. The interference of odd harmonics generated at the front and at the back of the pulse will then lead to modification of harmonic lineshapes, within their width. However, it will not turn odd harmonics into even. If the contributions from the raising and the falling parts of the pulse are phase-shifted by *π* in some spectral window, strong reshaping and suppression in this spectral window may result. This is precisely what we find for the heaviest isotope, 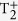, for a 20-cycle pulse, between harmonics 33–45: overall suppression, strong modification of line-shapes, but prominent odd harmonics nevertheless. To shift the harmonic lines on the scale of *ħω*_IR_, where *ω*_IR_ is the laser frequency, one has to modify the interference between successive half-cycles. We now show that the generation of a permanent dipole moment due to electron localization is the origin of such modification and the appearance of even harmonics.

## Electron Localization

**Figure 2:**
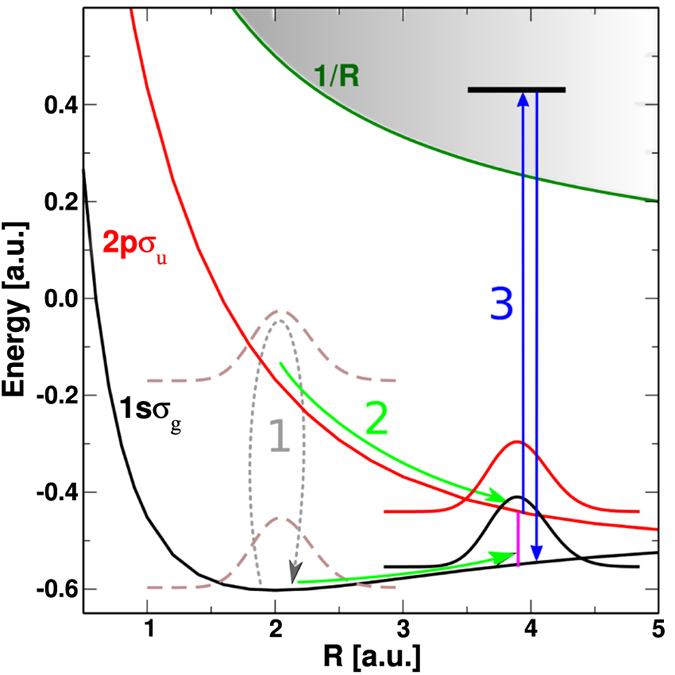
Sketch of the dynamics leading to electron localization. Full curves: potential energy curves of 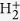. Dashed lines: initial nuclear wave packet. Vertical lines indicate transitions between molecular states. The meaning of processes 1, 2 and 3 is explained in the text.

The dissociative dynamics leading to eventual electron localization is well understood^28^. A sketch of this dynamics is shown in Fig. 2. During the first few cycles, the IR field, which is not yet intense enough to significantly ionize the molecule, induces Rabi-type oscillations between the 1*sσ*_*g*_ and 2*pσ*_*u*_ states, which lie much closer in energy than the 1*sσ*_*g*_ state and the ionization continuum (process 1). As a result, excited vibrational states associated with the 1*sσ*_*g*_ electronic state can be efficiently populated^30^, thus creating a vibrational wave packet. A similar vibrational wave packet is formed in the 2*pσ*_*u*_ state. These wave packets then move towards larger internuclear distances (process 2), until they reach a region of internuclear distances where the 1*sσ*_*g*_ and 2*pσ*_*u*_ electronic states are very close in energy and are strongly coupled by the IR field. Therefore they mix, leading to localized states 1*sσ*_*g*_ ± 2*pσ*_*u*_. The characteristic value of the internuclear distance *R* = *R*_*C*_ where the onset of localization occurs can be estimated by using the criterion from ref. 31, 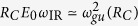, where *E*_0_ is the field amplitude and *ω*_*gu*_ the energy difference between the 1*sσ*_*g*_ and 2*pσ*_*u*_ states at *R* = *R*_*C*_. This criterion yields 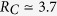 a.u., shown in Fig. 2 with a pink vertical line. Localization opens the barrier for dissociation, through the process called bond softening^32^. By then, the IR field has reached (or nearly reached) its peak intensity and tunnel ionization becomes prominent (process 3). This enhancement of tunnel ionization in the long *R* region is caused by both the reduced ionization potential (compared to the equilibrium geometry), which decreases with *R*, and the enhanced ionization caused by electron localization^33^,^34^. It is important to stress that localization of the electron on one or the other side of the molecule depends on the phase of the laser field^35^ and, therefore, can be controlled by the carrier-envelope phase^28^. Thus, the ulterior recombination process, i.e., harmonic generation, is acutely sensitive to the window of *R* where the medium symmetry is broken.

**Figure 3:**
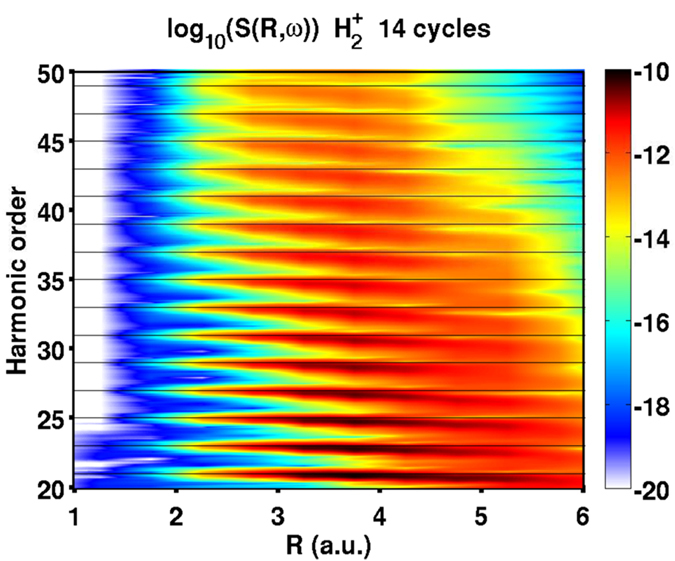
*R*-dependent HHG spectrum obtained from equation (1) for a pulse of 800 nm, *I* = 3 × 10^14^ W/cm^2^ and 14 optical cycles for 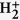. The horizontal lines indicate the position of odd harmonics.

To quantify this picture and confirm our explanation, we have evaluated an *R*-dependent HHG spectrum, S(R, ω), as the square of the Fourier transform of


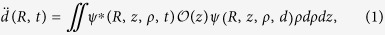


where *ψ* is the solution of the time-dependent Schrödinger equation (TDSE), *ρ* and *z* are cylindrical coordinates describing the position of the electron (see Methods), *R* is the internuclear distance, and 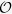 is the dipole acceleration operator. The calculated *R*-dependent HHG spectrum is shown in Fig. 3 for the 14-cycles pulse. As can be seen, even harmonics appear near the localization distance 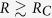 a.u., far beyond the equilibrium distance *R*_*eq*_ = 1.9 a.u., in agreement with our arguments. This also explains the lack of even harmonics for heavier isotopes, which dissociate slower and do not reach the localization region before the pulse is over.

**Figure 4:**
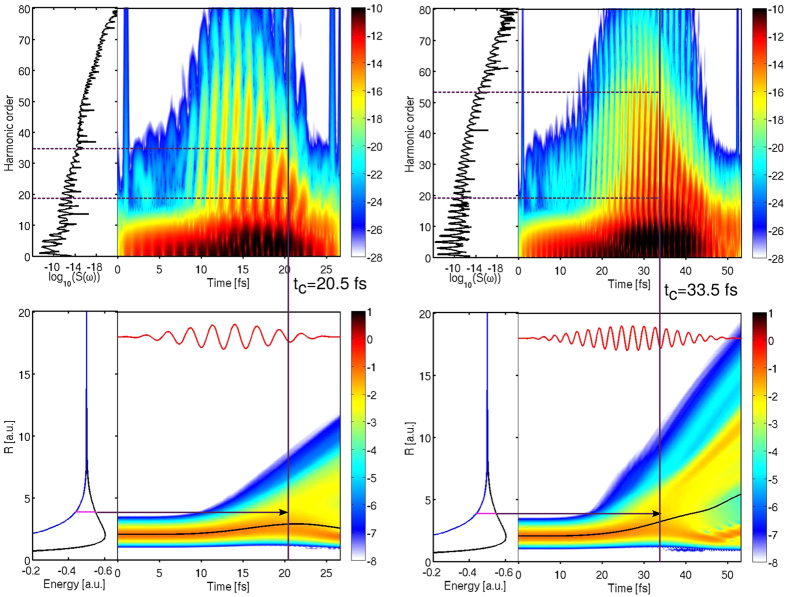
Gabor profile (top panels) and nuclear wave packet distribution (bottom panels) for a pulse of 800 nm, *I* = 3 × 10^14^ W/cm^2^ and 10 (left) and 20 (right) optical cycles for 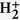. The corresponding HHG spectra are shown on the left of the top panels. The dashed lines in the top panels represent the ionization threshold (lower line) and the upper bound for the appearance of even harmonics. For clarity, the potential energy curves of the 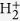 states are shown on the left of the bottom panels. The black horizontal arrow indicates the value of the internuclear distance at which electron localization occurs. The vertical lines that go from the upper to the lower panels represent the time *t*_*C*_ at which electron localization occurs (see text). The black curves in the lower panel represent the average *R* value of the nuclear wave packet.

Figure 4 shows the time-windowed Fourier transforms of the calculated wave functions (Gabor profiles) and the time evolution of the nuclear wave packets in 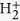 for 10 and 20 cycle pulses. For each pulse duration we select the time, *t*_*C*_, at which the density around the critical internuclear distance, *R*_*C*_ = 3.7 a.u., is largest. By looking at the Gabor profile at *t* > *t*_*C*_ and at the harmonics that are emitted at *t*_*C*_, we can predict the location of the even harmonics in the HHG spectra. For the 10-cycles pulse, *R*_*C*_ is reached when the intensity of the laser pulse is already decreasing. This leads to even harmonics in the lower region of the HHG spectrum. In contrast, for the 20-cycles pulse, *R*_*C*_ is already reached when the intensity of the laser pulse is still increasing. Consequently, even harmonics appear at higher energies in the spectrum. Note that even harmonics arise for sufficiently long pulses, when the nuclear wave packet has had enough time to reach *R*_*C*_. By controlling the pulse duration one thus controls the region of the plateau where even harmonics appear.

It is interesting to point out that the magnitude of the nuclear wave packets shown in Figure 4 outside the Franck-Condon region, e.g., at *R*~*R*_*C*_, is much larger than that obtained from earlier low-dimensionality models^20^. In other words, nuclear dynamics is actually much faster in the true three-dimensional space. Consequently, the intensity of the even harmonic peaks predicted by low dimensionality models is much lower than that of the odd harmonic peaks, which dominate the spectrum for all harmonic orders, in contrast with the results of the present work.

**Figure 5:**
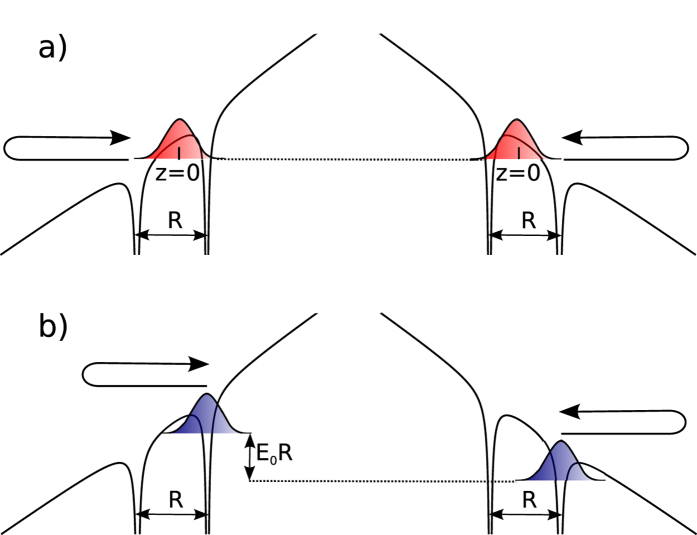
Sketch of the trajectories followed by the electron in two consecutive half cycles when it is initially delocalized (**a**) and localized (**b**).

The physical picture that identifies electron localization as the origin of even-harmonic generation is further substantiated by analyzing the phase-shift in the emission bursts during successive half-cycles, accumulated due to the induced dipole moment. Figure 5 shows a sketch of the typical trajectory followed by an electron starting in a delocalized and localized initial bound state. In the second case, the localized bound state experiences a linear Stark shift, leading to additional phase difference accumulated between the ionization *t*_*i*_ and recombination *t*_*r*_ times,


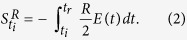


For trajectories generated in consecutive half-cycles, the accumulated phase difference is twice as large,


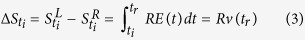


In the last equality we have used the three-step model of HHG: for the electron starting with zero velocity at *t*_*i*_, the integral of the laser field *E*(*t*) between *t*_*i*_ and *t*_*r*_ gives its instantaneous electron velocity *v*(*t*_*r*_) at the return time *t*_*r*_. Thus, we can rewrite this phase difference in terms of the electron recombination energy *E*_kin_ = *v*^2^(*t*_*r*_)/2, or the emitted photon energy, *Nω*_IR_,


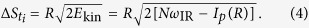


**Figure 6:**
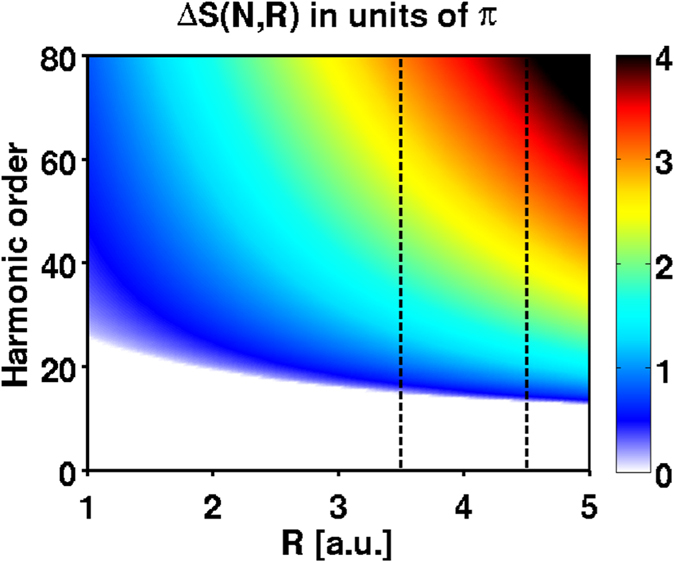
Additional phase difference between two consecutive half cycles for an electron initially localized on one side of the molecule (in units of *π*). The region between the vertical dashed lines indicates where localization actually takes place.

Figure 6 shows this phase difference as a function of the harmonic order and the internuclear distance. We have checked, by solving the classical equations of motion numerically, that the inclusion of the molecular potential adds an extra phase, which is however much smaller than that shown in Fig. 6, so that the global picture remains unchanged. In the vicinity of 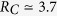 a.u., where localization takes place, there is a large range of harmonic orders where the additional phase difference between the right and the left trajectories is ≈*π*, thus leading to even harmonic generation. According to Fig. 6, this occurs approximately between harmonic orders 20 and 40. Remarkably, Fig. 6 predicts a phase difference of ≈3*π*, hence a revival of even harmonic emission, between harmonic orders 60 and 80, in excellent agreement with the appearance of even harmonics in the cut-off region predicted by the ab initio calculations for 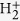 (see Fig. 1c,d). Finally, Fig. 6 also shows that the positions of the harmonic lines experience a slow frequency shift across the spectrum as *R*_*C*_ is varied, again in agreement with our ab-initio observations.

## Conclusion

Using the example of one-electron homonuclear diatomic molecules, we have shown how dynamics induced in the molecule can lead to a dramatic breakdown of the standard selection rules in high harmonic generation, including the nearly complete suppression of odd and the appearance of even harmonics for multi-cycle laser pulses, in a broad window of the harmonic spectrum. Our analysis links strong shifts of the harmonic lines with the appearance of a permanent dipole moment in dissociating homonuclear molecules, caused by electron localization. This dipole moment introduces phase shifts between the emission bursts during successive laser half-cycles, which can approach and exceed *π*. The ultimate origin of symmetry breaking is the sensitivity of the overall process to the carrier envelope phase of the laser pulse. The fact that minute changes in the driving laser field, associated with the carrier-envelope phase of a multi-cycle (20-cycle) laser pulse, can lead to strong effects in the harmonic spectrum, reflects very strong sensitivity of the underlying dissociation – localization dynamics to the details of the driving field. In classical systems, such extreme sensitivity is characteristic of dynamical chaos. Thus, our results suggest that high harmonic generation might also be a sensitive probe for the onset of dynamical chaos in light-driven systems.

**Figure 7:**
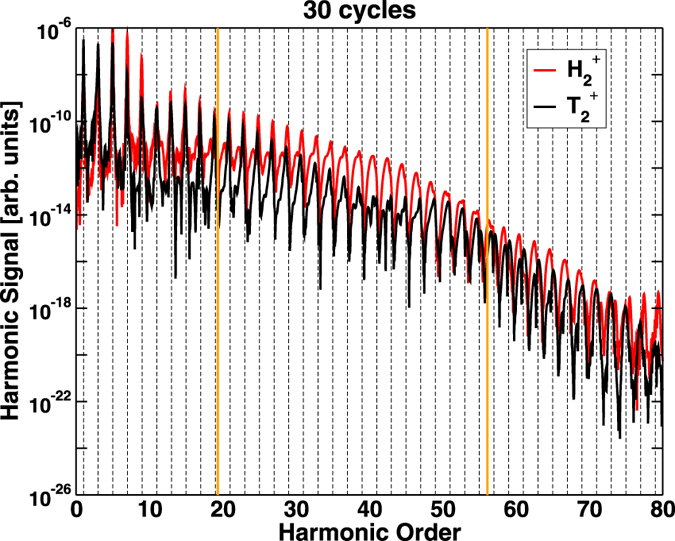
HHG spectrum for a pulse of 800 nm, *I* = 3 × 10^14^ W/cm^2^ and 30 optical cycles for 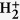 and 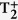. The dashed vertical lines indicates odd harmonics. The thick vertical lines indicate the ionization threshold (left line) and the cut-off energy obtained from *E*_cutoff_ = *I*_*p*_ + 3.17*U*_*p*_ (right line).

At first sight, the pulse durations required to see dominant even-harmonic emission are much longer than what one would normally expect when studying electron localization by detecting dissociating fragments. Electron localization, when observed in molecular fragmentation, is a ‘zero background’ signal, i.e., with no contamination from non dissociative channels. In this case, the shorter the laser pulse, the better, since asymmetry in the pulse temporal profile due to the carrier envelope phase (CEP) matters. This is why pioneering experiments on electron localization in molecular fragmentation^7^,^28^ have been most successful for relatively short pulses. However, this is not the case for HHG, where non-dissociated molecules also contribute to the signal. For short pulses, when the fraction of dissociating molecules is still tiny, their response and the associated even harmonic signal drown in the response of the non-dissociating molecules. Only when the pulses are long enough to produce a significant number of dissociating molecules, emission of even harmonics will be observed. On the other hand, if the pulse is too long, the effect should disappear, because the CEP does not matter any more and, therefore, the probabilities to localize the electron on the left or on the right of the dissociating molecule are identical. Clearly, there must be a window of pulse durations for even harmonics in HHG. Calculations performed for 30-cycle pulses show that this is indeed the case: as can be seen in Fig. 7, for such a long pulse, one begins to see a decrease in the strength of the even harmonic signal.

One could also ask whether the effect we observe would still be present in the macroscopic response. First, we note that as far as initial conditions are concerned, we start with the vibrational ground state. This is adequate for 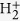 even at room temperature. As far as the phase matching is concerned, our analysis shows nothing special regarding the phases of even harmonics compared to those of the odd ones. Thus, we do not see any reason for the propagation to quench the single-molecule effects.

## Methods

Our theoretical method has been described in detail in ref. 36. Briefly, we solve the three-dimensional (3D) time-dependent Schrödinger equation (TDSE) in cylindrical coordinates, *ρ* and *z* for the electron, *R* for the internuclear distance, where *z* coincides with the linear polarization direction of the electric field. We assume that the molecules are aligned with the linearly polarized driving IR field, neglecting their rotations. The electron azimuthal coordinate *ϕ* is removed by the cylindrical symmetry of the problem. The TDSE reads


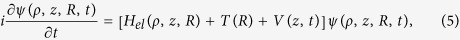


where *H*_*el*_ = *T*_*el*_ + *V*_*eN*_ + 1/*R* is the electronic Hamiltonian of 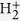 or its molecular isotopes, *T* is the nuclear kinetic energy operator, *V* = *zE*(*t*) describes the interaction with the laser field in the length gauge, *T*_*el*_ is the nuclear kinetic energy operator, and the Coulomb potential *V*_*eN*_ describes the electron – nuclei interaction. Atomic units are used throughout unless stated otherwise. The external laser field is *E*(*t*) = *E*_0_*f*(*t*)sin(*ωt*), where


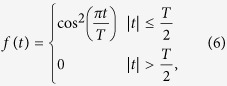


*E*_0_ the electric field amplitude, *ω* the central frequency corresponding to the wavelength *λ* = 800 nm, and *T* the total pulse duration ranging between 5 and 20 optical cycles (13.34–53.36 fs). The peak intensity in all calculations is 3 × 10^14^ W/cm^2^.

We have used a non-equidistant cubic 3D grid with |*z*| < 55, *ρ* < 50 and *R* < 30, and grid spacings Δ*z* = 0.1, Δ*ρ* = 0.075 and Δ*R* = 0.05 a.u. at the center of the grid. The grid spacings increase gradually from the origin to the box boundaries^36^. The Crank-Nicolson propagator with a split-operator method was used for time-propagation, with a time step Δ*t*_*elec*_ = 0.011 a.u. for the electrons and Δ*t*_*nuc*_ = 0.11 a.u. for the nuclei. The convergence of these parameters was checked. The initial, ground, state of the 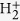 molecule was obtained by diagonalizing the unperturbed Hamiltonian using the SLEPc routines^37^. Absorbers were placed at |*z*| > 35 a.u. and *ρ* > 30 a.u. to avoid artificial reflections from the boundaries.

At each time step, we have calculated the time-dependent dipole 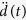 as


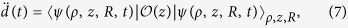


where 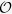 is the dipole acceleration operator and integration is performed over electronic (*ρ*, *z*) and nuclear (*R*) coordinates. The harmonic spectrum 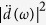 is given by the square of the Fourier transform (FT) of 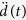.

## Additional Information

**How to cite this article**: Silva, R. E. F. *et al*. Even harmonic generation in isotropic media of dissociating homonuclear molecules. *Sci. Rep.* **6**, 32653; doi: 10.1038/srep32653 (2016).

## References

[b1] Krausz F. & Ivanov M. Attosecond physics. Rev. Mod. Phys. 81, 163–234 (2009).

[b2] Scrinzi A., Ivanov M. Y., Kienberger R. & Villeneuve D. M. Attosecond physics. J. Phys. B: At. Mol. Opt. Phys. 39, R1 (2006).

[b3] Christov I. P., Murnane M. M. & Kapteyn H. C. High-harmonic generation of attosecond pulses in the “single-cycle” regime. Phys. Rev. Lett. 78, 1251 (1997).

[b4] Sansone G. . Isolated single-cycle attosecond pulses. Science 314, 443–446 (2006). 17053142 10.1126/science.1132838

[b5] Popmintchev D. . Ultraviolet surprise: Efficient soft x-ray high-harmonic generation in multiply ionized plasmas. Science 350, 1225–1231 (2015). 26785483 10.1126/science.aac9755

[b6] Lépine F., Ivanov M. Y. & Vrakking M. J. Attosecond molecular dynamics: fact or fiction? Nature Photonics 8, 195–204 (2014).

[b7] Sansone G. . Electron localization following attosecond molecular photoionization. Nature 465, 763–766 (2010). 20535207 10.1038/nature09084

[b8] Smirnova O. . High harmonic interferometry of multi-electron dynamics in molecules. Nature 460, 972–977 (2009). 19626004 10.1038/nature08253

[b9] Cavalieri A. L. . Attosecond spectroscopy in condensed matter. Nature 449, 1029–1032 (2007). 17960239 10.1038/nature06229

[b10] Schiffrin A. . Optical-field-induced current in dielectrics. Nature 493, 70–74 (2013). 23222521 10.1038/nature11567

[b11] Ghimire S. . Observation of high-order harmonic generation in a bulk crystal. Nature Physics 7, 138–141 (2011).

[b12] Calegari F. . Ultrafast electron dynamics in phenylalanine initiated by attosecond pulses. Science 346, 336–339 (2014). 25324385 10.1126/science.1254061

[b13] Kraus P. . Measurement and laser control of attosecond charge migration in ionized iodoacetylene. Science 350, 790–795 (2015). 26494175 10.1126/science.aab2160

[b14] Corkum P. B. Plasma perspective on strong field multiphoton ionization. Phys. Rev. Lett. 71, 1994–1997 (1993). 10054556 10.1103/PhysRevLett.71.1994

[b15] Kulander K. J. & Schafer K. Super-Intense Laser-Atom Physics (NATO ASI Series, 1993).

[b16] Lewenstein M., Balcou P., Ivanov M. Y., L’Huillier A. & Corkum P. B. Theory of high-harmonic generation by low-frequency laser fields. Phys. Rev. A 49, 2117–2132 (1994). 9910464 10.1103/physreva.49.2117

[b17] Kuchiev M. Y. Atomic antenna. JETP Lett 45, 404–406 (1987).

[b18] Dahlström J., L’Huillier A. & Mauritsson J. Quantum mechanical approach to probing the birth of attosecond pulses using a two-colour field. J. Phys. B: At. Mol. Opt. Phys. 44, 095602 (2011).

[b19] Lein M. Attosecond probing of vibrational dynamics with high-harmonic generation. Phys. Rev. Lett. 94, 053004 (2005). 15783636 10.1103/PhysRevLett.94.053004

[b20] Morales F. . High harmonic spectroscopy of electron localization in the hydrogen molecular ion. J. Phys. B: At. Mol. Opt. Phys. 47, 204015 (2014).

[b21] Riviére P. . Time reconstruction of harmonic emission in molecules near the ionization threshold. J. Phys. B: At. Mol. Opt. Phys. 47, 241001 (2014).

[b22] Bian X.-B. & Bandrauk A. D. Probing nuclear motion by frequency modulation of molecular high-order harmonic generation. Phys. Rev. Lett. 113, 193901 (2014). 25415907 10.1103/PhysRevLett.113.193901

[b23] Bandrauk A. D., Chelkowski S. & Lu H. Quantum Dynamic Imaging: Theoretical and Numerical Methods, chap. Correlated electron-nuclear motion visualized using a wavelet time-frequency analysis, 23 (Springer, 2011).

[b24] Bandrauk A. D., Chelkowski S. & Lu H. Signatures of nuclear motion in molecular high-order harmonics and in the generation of attosecond pulse trains by ultrashort intense laser pulses. J. Phys. B: At. Mol. Opt. Phys. 42, 075602 (2009).

[b25] Kreibich T., Lein M., Engel V. & Gross E. K. U. Even-harmonic generation due to beyond-born-oppenheimer dynamics. Phys. Rev. Lett. 87, 103901 (2001). 11531478 10.1103/PhysRevLett.87.103901

[b26] Du H., Yue S., Wang H., Wu H. & Hu B. Reexamining the high-order harmonic generation of hd molecule in non-born-oppenheimer approximation. J. Chem. Phys. 144, 114308 (2016). 27004877 10.1063/1.4943371

[b27] Bredtmann T., Chelkowski S. & Bandrauk A. D. Monitoring attosecond dynamics of coherent electron-nuclear wave packets by molecular high-order-harmonic generation. Phys. Rev. A 84, 021401 (2011).

[b28] Kling M. . Control of electron localization in molecular dissociation. Science 312, 246–248 (2006). 16614216 10.1126/science.1126259

[b29] Smirnova O., Spanner M. & Ivanov M. Anatomy of strong field ionization ii: to dress or not to dress? J. Mod. Opt. 54, 1019–1038 (2007).

[b30] Silva R. E. F., Catoire F., Rivière P., Bachau H. & Martín F. Correlated electron and nuclear dynamics in strong field photoionization of H_2_^+^. Phys. Rev. Lett. 110, 113001 (2013). 25166527 10.1103/PhysRevLett.110.113001

[b31] Ivanov M. Y., Corkum P. & Dietrich P. Coherent control and collapse of symmetry in a two-level system in an intense laser field. Laser Phys. 3, 375–380 (1993).

[b32] Bucksbaum P. H., Zavriyev A., Muller H. G. & Schumacher D. W. Softening of the H_2_^+^ molecular bond in intense laser fields. Phys. Rev. Lett. 64, 1883 (1990). 10041519 10.1103/PhysRevLett.64.1883

[b33] Zuo T. & Bandrauk A. D. Charge-resonance-enhanced ionization of diatomic molecular ions by intense lasers. Phys. Rev. A 52, R2511 (1995). 9912637 10.1103/physreva.52.r2511

[b34] Seideman T., Ivanov M. Y. & Corkum P. B. Role of electron localization in intense-field molecular ionization. Phys. Rev. Lett. 75, 2819 (1995). 10059413 10.1103/PhysRevLett.75.2819

[b35] Kelkensberg F., Sansone G., Ivanov M. Y. & Vrakking M. A semi-classical model of attosecond electron localization in dissociative ionization of hydrogen. Phys. Chem. Chem. Phys. 13, 8647–8652 (2011). 21483973 10.1039/c1cp20058e

[b36] Niederhausen T., Thumm U. & Martín F. Laser-controlled vibrational heating and cooling of oriented H_2_^+^ molecules. J. Phys. B: At. Mol. Opt. Phys. 45, 105602 (2012).

[b37] Campos C. . *SLEPc Users Manual* (2011). URL http://www.grycap.upv.es/slepc/.

